# Assessment of the Welfare of Experimental Cattle and Pigs Using the Animal Welfare Assessment Grid

**DOI:** 10.3390/ani11040999

**Published:** 2021-04-02

**Authors:** Molly Ryan, Ryan Waters, Sarah Wolfensohn

**Affiliations:** 1 Faculty of Health and Medical Sciences, University of Surrey School of Veterinary Medicine, VSM Building, Daphne Jackson Rd, Guildford GU2 7AL, UK; s.wolfensohn@surrey.ac.uk; 2 The Pirbright Institute, Ash Road, Woking, Surrey GU24 0NF, UK; ryan.waters@pirbright.ac.uk

**Keywords:** welfare assessment, AWAG, cattle, pigs, harm, benefit

## Abstract

**Simple Summary:**

The Animal Welfare Assessment Grid is a method for assessing quality of life, originally designed for experimental primates. This study adapts this welfare assessment tool for use in cattle and pigs, by adapting the factors included in welfare assessment for these species and including data which had been collected previously as the standard approach to monitoring these species in a research setting. The main intention is that the results presented will demonstrate how the data collected in a research environment can be improved for welfare assessment and also demonstrate the applicability of this welfare assessment tool to cattle and pigs. This paper emphasises the importance of including behavioural information when assessing welfare and not simply relying on assessment of physical condition. As a tool for assessing quality of life over a lifetime, the Animal Welfare Assessment Grid also demonstrated the potential for aiding the decision-making of when euthanasia should be performed.

**Abstract:**

The Animal Welfare Assessment Grid (AWAG) is a method for assessing quality of life, originally designed for experimental primates. This study adapts the AWAG for use in cattle and pigs, by adapting the factors included for these species and including data which had been collected previously as the standard approach to monitoring these species in research. The intention is that the results presented here will allow the future data collected for experimental cattle and pigs to be optimised for inclusion in an AWAG. Data were collected from two vaccine assessment studies at the Pirbright Institute. Factors were scored for every recorded event using retrospective data and CCTV clips. There was a lack of behavioural data recorded in both studies, which limited the accuracy of assessing each animal’s welfare. This paper emphasises the importance of including behavioural information when assessing welfare and not simply relying on assessment of physical condition. Scores peaked following an exponential rise as animals reached set humane end points. This demonstrated the potential of using the AWAG to aid the decision-making of when euthanasia should be performed. Our study shows the AWAG to be a useful tool for assessing welfare, which can be used in harm:benefit assessment.

## 1. Introduction

The animal welfare assessment grid (AWAG) has been developed as a tool to measure an animal’s level of welfare using ‘cumulative lifetime experience’ [1], which supports the requirement in the EU directive 2010/63, that the use of animals in scientific research should take into account the lifetime experience of the individual animal, and the ‘cumulative suffering’ within procedures [2].

Many animal welfare assessment tools have been reported but most only look at a snapshot in time [3,4,5]. Animal welfare can be considered using a variety of frameworks including:

- The five freedoms (freedom from hunger and thirst; freedom from discomfort; freedom from pain, injury, and disease; freedom to express normal behaviour; and freedom from fear and distress) [6,7].
- The five needs (access to fresh water and a suitable diet that will keep them healthy; adequate shelter and somewhere comfortable to rest; access to veterinary treatment, but also steps taken to prevent pain, injury, or disease; company of other animals of their own kind with enough space and proper facilities so they can behave in a natural way, to be kept in conditions that mean they will not suffer; and to be treated in a way that does not frighten or distress them) [8].
- The five domains (nutrition, environment, health, behaviour and mental state) [9].

These frameworks are essentially quite similar and reflect the fact that animal welfare is considered to constitute more than the basic clinical condition and health status of an animal—it must also include their mental and emotional condition [10,11]. An animal can have good physical health and meet the standards of a ‘healthy’ animal for research, and still have poor welfare. For example, a cow housed indoors in a small area with no enrichment may have normal physiological parameters, and be free from infectious diseases, but have a compromised quality of life due to the inability to produce a normal range of behaviours as a result of the relatively barren environment in which it lives. Therefore, an accurate assessment of welfare needs to take into account the physical condition of the animal, its psychological condition and behaviour, the quality of the environment in which it is kept, and any procedures that take place on that animal even if they are being done for the animal’s benefit in the longer term.

Various attempts at recording an animal’s welfare have been made, however most only deal with a single point in time and therefore cannot record the lifetime experiences of the animal [12]. The Farm Animal Welfare Council has split quality of life into three broad categories; a life not worth living; a life worth living; and a good life. Assessment should be based on the number and proportion of ‘positive emotional states or experiences’, for example comfort and interest, to ‘negative emotional states or experiences’, for example pain or boredom. A ‘Good Life’ is defined as a ‘life that is over and above that of a mere life worth living’ and involves an ‘especially high affective ratio of positively to negatively valued experiences’ [13].

The Animal Welfare Assessment Grid is composed of a cruciform grid with X- and Y- axes. Each arm of the X- and Y- axes represent a different scored parameter [14]. The 4 parameters are as follows:

- Physical: This reflects an animal’s clinical state of health including factors such as body condition, clinical signs of disease and lameness.
- Behavioural/Psychological: This reflects an animal’s psychological condition, and includes factors such as abnormal/stereotypic behaviour, expression of natural behaviours and social structure.
- Environmental: This reflects the animal’s housing, including factors such as lighting, flooring, enclosure complexity and enrichment provision.
- Procedural: This assesses the challenge to the animal arising from experimental events and clinical/husbandry events, and includes factors such as blood sampling, clinical examination, vaccination and sedation [14].

**Figure 1:**
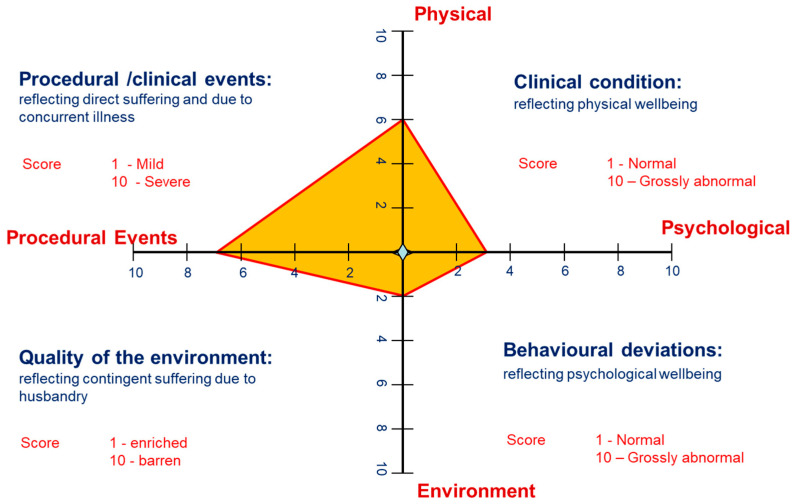
An example of the AWAG showing the four parameters scored on the cruciform grid. In this example, the scores for each parameter are as follows: physical 6; psychological 3; procedural events 7; environment 2. The cumulative welfare assessment score would be calculated from the area of the grid.

An example of the assessment grid can be seen in Figure 1. Within each parameter, welfare factors relevant to the species are chosen for assessment [1]. Each factor is scored from one to ten: a score of 1 is considered the ‘gold standard’ and is applicable to animals receiving gold-standard care in a non-experimental, domesticated environment. The further the animal scores away from 1, to a maximum of 10, the further the animal’s welfare is considered to deviate from the optimum ‘gold standard’. The average of these factors gives a score for each parameter, and this is scored on the cruciform grid as shown in Figure 1. The area of the grid is calculated to give an objective score of the animal’s welfare state at that point in time, this is known as the Cumulative Welfare Assessment Score (CWAS). The CWAS scores for each lifetime event (including every procedure carried out within the study, and any contingent events such as transport or injury) can then be plotted on a graph to show an animal’s welfare over a lifetime, an example of this can be seen in the results section.

This system was originally designed for non-human primates in a research environment [14] and has been shown to allow the ‘critical evaluation of animals’ quality of life and the recognition of signs of poor welfare, such that improvement strategies may be selected’ [1]. Welfare assessment should take into consideration the range of biological requirements and needs of an animal which are specific for each species. There is therefore no ‘one size fits all strategy’, but the AWAG has been successfully applied to primate species housed in zoos and to other species, including large felids (namely Amur tiger (*Panthera tigris altaica*), Amur leopard (*Panthera pardus orientalis*), snow leopard (*Panthera uncial*) and cheetah (*Acinonyx jubatus*)), scimitar-horned oryx (*Oryx dammah)* and giraffe (*Giraffa camelopardalis*) [15]. There is a requirement to assess whether the AWAG can be successfully applied to farm species in addition to non-human primates and zoo species.

This paper reports on the success of applying the AWAG to the assessment of welfare of cattle (*n* = 7) and pigs (*n* = 6) used in an infectious disease research environment. In particular, the AWAG has been used to take into account non-procedural events (for example, transport and injuries incurred not as a result of the study) when assessing an animal’s quality of life, as opposed to just those resulting from the regulated procedures. The harm:benefit assessment of projects involving research using animals focuses on harms related to the regulated procedures carried out on the animals—‘direct’ suffering. However, the ‘contingent’ suffering should also be evaluated and considered which includes suffering incurred not as a direct result of the scientific study, such as transport to the research institute, housing, and non-procedural health issues [16]. It is predicted that factoring in the contingent, non-procedural events for cattle and pigs used in infectious disease research will significantly alter the overall assessment of an animal’s welfare compared to direct suffering alone, as has been shown in previous studies [1]. This paper is expected to highlight the need for improvement and show the bias in research towards recording data relevant to the study, as opposed to data relevant to assessing animal welfare. The other outcome from using retrospective data to populate the AWAG will be to help identify factors which could be added to the current “on-study” data collection templates, to enhance the welfare assessment of farm animals in research.

## 2. Materials and Methods 

### 2.1. Retrospective Studies Used for Analysis

The data used to populate the AWAG in this manuscript were provided retrospectively from a series of animal studies at The Pirbright Institute. These studies were approved by the Animal Welfare Ethical Review Board (AWERB) of The Pirbright Institute and carried out under project licenses in line with the Animals (Scientific Procedures) Act 1986, approved by the Home Office. These were vaccine efficacy studies carried out using standard commercial pig and cattle breeds, examining African Swine Fever (ASF) vaccines and Foot-and-Mouth Disease (FMD) vaccines, respectively. Animals were sourced from a standard agricultural environment, adhering to the health monitoring principles of the FELASA guidelines for the health management of ruminants and pigs [17], and held in accordance with the Home Office Code of Practice [18].

During the course of each study, all animals were challenged with pathogenic virus a period of time following vaccination. Pre-infection data during the acclimatization period was available and was scored for both species before they were exposed to disease. This included scoring the environment in which they were in, their physical condition on arrival at the research institute, and the behavioural/emotional state of each animal during the acclimatization period. The behavioural state was scored based on a tick box selection (normal-aggressive-nervous-dull/depressed) carried out contemporaneously by the animal care technicians within the first 24 h after arrival.

The animals were transported to the research facility, where the cattle and pigs were allowed to acclimatise for at least 5 and 6 days, respectively before the first regulated procedures were carried out. The animals remained in the same environment for the duration of the study and were not moved.

On day 0 of the study, cattle were vaccinated via an intramuscular injection, their rectal temperatures recorded, and blood samples taken. They were all challenged with virulent FMDV into the dermis of the tongue epithelium under sedation on day 21. Each day following challenge the cattle were observed for clinical signs associated with FMDV infection and data recorded on FMD specific clinical observation scoring. The data collected included rectal temperatures, signs of anorexia, ptyalism, nasal discharge and lameness. All feet were inspected daily for the presence of lesions. All cattle were euthanised when they either reached the humane end points set by the study (details of which can be found in the Appendix A), the scientific endpoints (observation of vesicular lesions), or on day 8 post-challenge, whichever was sooner. Day 8 post-challenge represented the end of the study.

**Figure 2:**
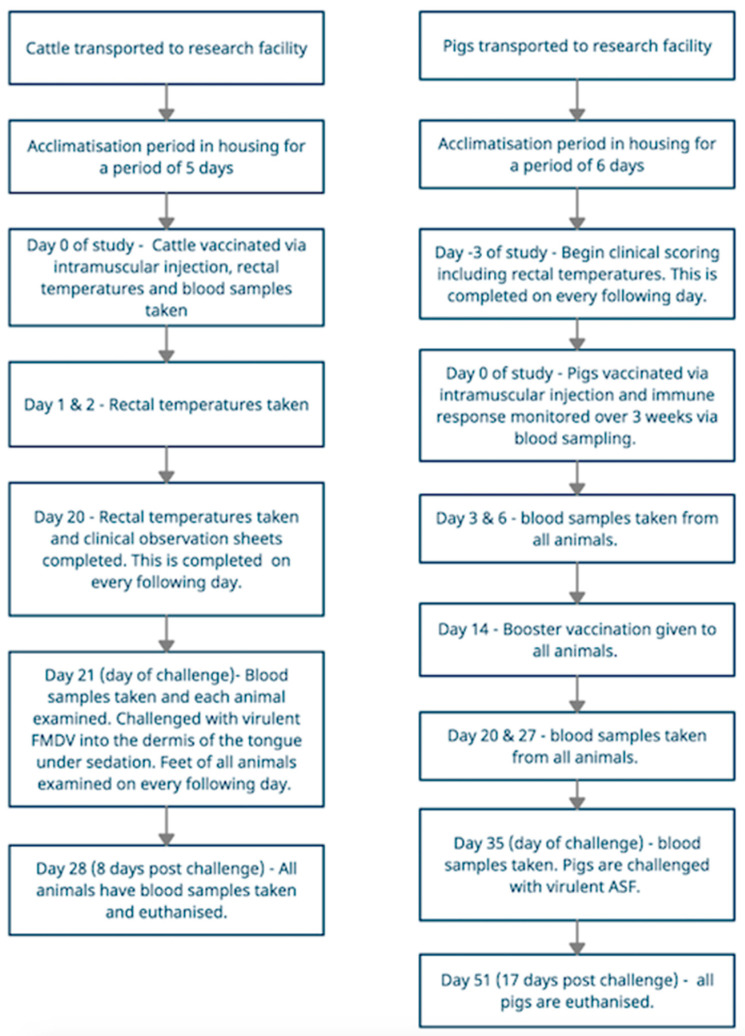
Flow charts showing the procedural events for cattle infected with Foot and Mouth Disease, and pigs infected with African Swine Fever. Animal welfare assessment grids were completed for every event taking place.

Pigs were vaccinated via an intramuscular injection on day 0 of the study and antibody responses were monitored over 3 weeks by assessing blood samples. If minimum immune response was detected, they were challenged with the virulent ASF virus. Following the initial vaccination, animals were observed at least once daily and clinical signs, including temperature, were recorded on an ASF specific clinical observation scoring sheet. All pigs were euthanised either on reaching the humane end points (which were the same as the scientific end-points) set by the study (details of which can be found in the Appendix A), or on day 17 post-challenge, whichever was sooner. Day 17 post challenge represented the end of the study. Both species were euthanised via an anaesthetic overdose followed by confirmation of cessation of circulation using auscultation. The sequence of events for both animal studies can be seen below in Figure 2.

### 2.2. Choosing Factors

Within each parameter of the Animal Welfare Assessment Grid (AWAG), factors were chosen relevant to the species and study being carried out. Some factors are applicable to many species regardless of the setting, for example food intake, and these were scored in both studies, but it is necessary to adapt some factors for the species and the study they are taking part in [14]. These were determined based on knowledge of the species [19,20], findings from previous studies (for example, the importance of enrichment provision) [1,21] as well as the clinical signs likely to be seen in animals infected with ASF or FMD [22,23]. Assessment of clinical condition therefore included lameness, lethargy, food intake, pyrexia, diarrhoea, nasal discharge, salivation and foot and mouth lesions. The full list of factors chosen and the reasoning for selection can be seen in Table 1. 

These factors were selected prior to viewing the retrospective data from each study, and so were not influenced by what data was available. Once decided upon, each factor was given a descriptor regarding what qualifies as scoring numbers one through ten (the full list of these can be seen in the Appendix A). This was performed by two researchers for inter-observer reliability using previously published studies involving use of the AWAG as a guide [1]. Once these criteria were set and defined as objectively as possible, scores for each factor were assigned to each animal for each lifetime event. The AWAG does rely on some subjective assessment by the researchers, however this is limited as much as possible by the use of set criteria tables which have been agreed and defined by more than one party. As examples, food intake and response to restraint for both cattle and pigs were scored as follows:

- Food intake:
  ○ Score 1—eating normally.
  ○ Score 2—Food intake slightly lower than normal for one day or animal reported hungry for 1 day.
  ○ Score 3—Food intake significantly lower than normal for one day or reported hungry for 2–3 days.
  ○ Score 4—Food intake slightly lower than normal for 2 days (<80%) or reported hungry for 4–5 days.
  ○ Score 5—Food intake significantly lower for 2 days (<50%) or reported hungry for 8–9 days.
  ○ Score 6—Food intake slightly lower than normal for 3 days (<80%) or reported hungry for 8–9 days.
  ○ Score 7—Food intake significantly lower than normal for 3 days (lower than 50%) or reported hungry for >9 days.
  ○ Score 8—Not eaten for 1 day
  ○ Score 9—Not eaten for 2 days
  ○ Score 10—Not eaten for 3 days.
- Response to restraint:
  ○ Score 1—Animal completely habituated, and restraint has no effect on animal’s behaviour.
  ○ Score 2—Animals have minimal response to restraint and show no stress. Animals are well habituated.
  ○ Score 3—Animals have moderate response to restraint but show no stress and are well habituated.
  ○ Score 4—Noticeable change in animal’s behaviour in response to restraint. Mild signs of stress/fear.
  ○ Score 5—Distinct change in animal’s behaviour. Moderate signs of stress/fear.
  ○ Score 6—Animal is noticeably stressed/scared +/− mild aggression.
  ○ Score 7—Animal shows elevated signs of stress/fear +/− moderate aggression.
  ○ Score 8—Animal shows elevated signs of stress/fear with significant aggression.
  ○ Score 9—Animal shows severe signs of stress/fear.
  ○ Score 10—Animal extremely scared and/or aggressive in response to restraint, with potential to cause danger to themselves or the keepers.

### 2.3. Data Collection

Using the data collected from each individual animal during the course of the studies, the online AWAG software was used to score each animal every time an event had taken place using the set criteria agreed by the researchers. The records used included the clinical score sheets for each animal, the statutory record of regulated procedures carried out, the animal request form (detailed study plan) and on farm/transport history. Short CCTV clips, recorded within the animal’s housing units, were also viewed for each species and used to assist scoring of behaviour, enrichment use, and housing. Behavioural factors were scored subjectively using the criteria found in the Appendix A, through CCTV and the limited data recorded during each study, which included a tick-box selection of normal/aggressive/nervous/dull-depressed on the animals’ arrival. Transport was scored using the same criteria in the Appendix A for housing and the expression of natural behaviour. It was expected that this score would be relatively high for transport using these criteria, however the main aim of scoring transport was to highlight the impact contingent events have on negatively or positively influencing an animal’s lifetime experience. Once scored, the software then produced cruciform grids for each animal’s welfare during that event. The Cumulative Welfare Assessment Scores (CWAS) calculated from the area of these grids were then plotted on a line graph, showing welfare across the entire period of data recording for each animal. Using this system allows for an assessment of cumulative welfare across an animal’s lifetime.

### 2.4. Data Analysis

The CWAS line graphs produced for lifetime experience identified parts of the study which had the biggest impact on welfare, and these could be further evaluated through analysing the individual cruciform grids for each event.

## 3. Results

### 3.1. Ease of Scoring Factors with the Data Available from a Standard Approach to Monitoring in a Research Setting 

The ease of scoring each factor accurately in cattle and pigs varied greatly. This was due to a lack of retrospective data in these areas and highlighted where recording of information could be better targeted for welfare assessment and use in the AWAG. This is illustrated in Table 2, which shows the bias given towards data collection for factors relevant to the scientific study, as opposed to data needed to assess animal welfare accurately.

### 3.2. Contingent Events and the Importance of Behaviour Assessment

**Figure 3:**
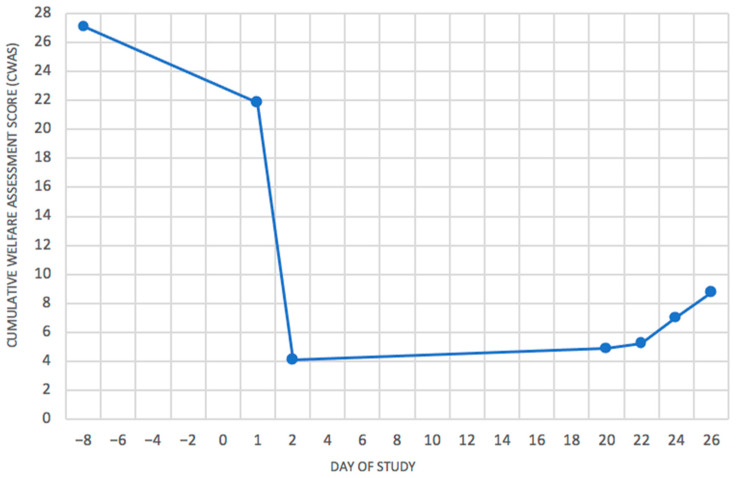
Cumulative welfare Assessment Score (CWAS) Timeline for a vaccinated bovine starting from Day −8. The cumulative welfare assessment scores are calculated from the area of the animal’s cruciform grids for each event. These are then plotted on the graph to display welfare over the animal’s lifetime. This demonstrates the impact transport (Day −8) had on welfare, as well as the effect of infection with FMD on Day 22.

The biggest change in welfare, secondary only to clinical disease, for all animals was found to be following transportation. An example of this can be seen in Figure 3, which shows the CWAS graph for a bovine. Transport to the research institute (day-8) had a significant impact on welfare with an overall score of 28.7, which dropped to 4.13 after the settling in period. Transport is not classed as a ‘study procedure’ but a contingent event, along with things such as injury or fighting within the group. Figure 3 clearly displays the effect this can have on welfare and demonstrates the importance of considering non-procedural events when assessing welfare, as well as procedural ones. This animal also began experiencing clinical signs of FMD on day 22 which began to impact welfare from then on. One of the main advantages of the AWAG is the ability to view welfare over a lifetime in visual form, and this is clearly demonstrated by Figure 3.

**Figure 4:**
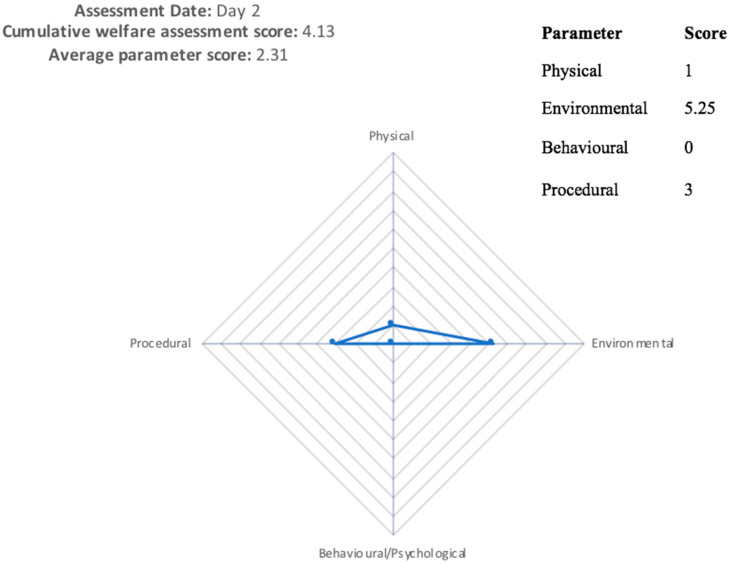
Cruciform grid displaying welfare assessment for a bovine on day 2, undergoing clinical examination (including rectal temperature), showing a cumulative welfare assessment score (CWAS) of 4.13 when behavioural data were unavailable. This CWAS score is calculated from the area of the grid.

**Figure 5:**
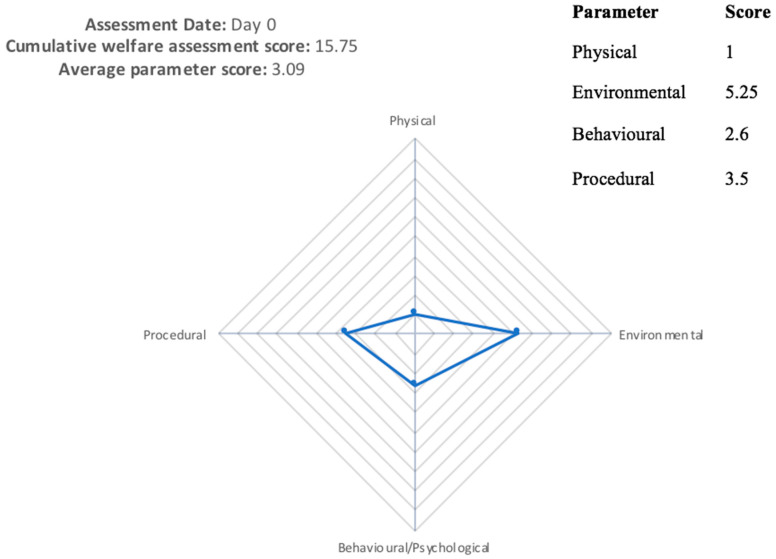
Cruciform grid showing welfare assessment for a bovine on day 0 undergoing blood sampling, showing a cumulative welfare assessment score (CWAS) of 15.75 when behavioural data were available. This CWAS score is calculated from the area of the grid.

Due to a lack of behavioural data recorded during the study, the true impact of the viral infection is likely to be far greater than is scored on the CWAS graph, demonstrating the need for recording behavioural/psychological factors, as can be seen from Figure 4 and Figure 5. In Figure 4, no behavioural data were available, and the animal receives a cumulative welfare score of 4.13. Figure 5 shows the same animal when behavioural data were available, scoring 15.75. Missing out this information greatly reduces the usefulness of scoring, as an animal which is well habituated to procedures will have a vastly different experience to an animal that is nervous of/aggressive towards human interaction [25].

### 3.3. Potential Application for Aiding the Decision of the Timing of Euthanasia

**Figure 6:**
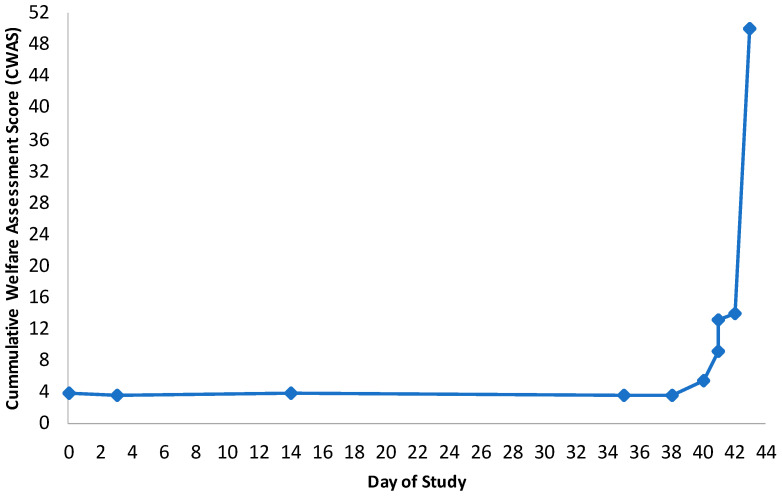
Cumulative Welfare Assessment Score (CWAS) timeline for porcine A, demonstrating the deterioration of welfare from being challenged with ASF on day 35, to requiring humane euthanasia on day 43. The cumulative welfare assessment scores are taken from the animal’s cruciform grids for each event. These are then plotted on the graph to display welfare over the animal’s lifetime.

Scoring also demonstrated the potential of the AWAG for aiding the decision of when euthanasia should take place, implementing the humane end point. An example of this can be seen in Figure 6, which clearly shows the deterioration of porcine A after challenge with African Swine Fever on day 35. A major benefit of the CWAS timeline is the ability to view welfare changes visually. Figure 6 is a good example of how the AWAG can be used to clearly highlight welfare changes so that they can be easily interpreted as either deteriorating or improving. This animal’s welfare deteriorated until it reached the project license humane end points, scoring 50.00 on the AWAG CWAS at that point in time, and requiring euthanasia on day 43.

## 4. Discussion

Physical, environmental and procedural factors could all be scored relatively accurately in these research animals for which the experiments they were involved in had a particular focus on clinical condition. It is clear that some factors are far easier to accurately assess and score than others, however this is due to a lack of available data and emphasises the need for recording a broader range of information during research. Behavioural factors need to be focused on, in future, if welfare assessment is going to be accurate. Effects on behaviour have a huge bearing on welfare and an effort should therefore be made for researchers to record these contemporaneously. As a minimum this should include the ability to express natural behaviour, the response to restraint/human interaction and use of enrichment. Transport had one of the largest detrimental effects on welfare, and although this was temporary, it demonstrates the need for assessing non-procedural contingent events with importance equal to the study’s procedural events.

### 4.1. This Paper Demonstrates the Bias Often Seen in Research towards Assessment of Factors Relevant to the Study, as Opposed to Including All Those Relevant to Assessing Welfare

The data collected in research is often mostly (if not exhaustively) related to assessing direct suffering on a snapshot basis. To some extent this is likely to be a result of the regulatory legal framework in place regarding procedural harms. This internal bias contributes to the difficulty in scoring contingent events, and this is demonstrated in Table 2. These have a significant effect on welfare, and as a result a conscious effort should be made to consider their impact [33,34].

The impact on natural behaviour, of clinical signs such as lethargy or lameness, is likely to be significant. As a key feature of welfare assessment, this is something that should be recorded in future scientific studies, to fully assess the welfare of the animals being used and ensure humane end points are set appropriately. The AWAG has the potential for linking with technology for telemetric monitoring of animal behaviours such as feeding and lameness. This data could be electronically accepted into the factors to be measured, and therefore used in the assessment of welfare. This would reduce the labour intensity of scoring behavioural factors, and potentially make behavioural assessment more practical in research environments [35].

Enrichment was provided in the form of straw and hay as a manipulable material for swine and also as a more natural feed source for cattle, respectively, and a feed ball hung from the ceiling for cattle. Use could not be assessed accurately in either species due to no available retrospective data, and limited CCTV footage. This could be something to add to study assessment sheets, which would provide information on not only the animal’s experience in the housing provided, but also whether the enrichment being provided is worthwhile, or whether alternatives should be considered. Different laboratory animal species will show preferences for different enrichment materials, and these can differ depending on many factors such as sex, age and group size. It is therefore important for a research institution to be able to evaluate whether the animals are using and benefiting from the enrichment provided in each individual study [36]. Some of the cattle could be seen interacting with the enrichment in one of the CCTV clips viewed, however, one clip alone cannot be generalised to how often the animals interacted with it over the course of the study. CCTV behavioural analysis is acknowledged as being labour intensive and is likely not practical to perform extensively in most research settings. Telemetric monitoring could be one method of consistently capturing this information.

### 4.2. Clinical Factors Had the Most Significant Impact on Welfare. This Is Expected in Animals Being Used for Infectious Disease Studies, although Importance May Be Skewed by the Data Available

Clinical factors due to the experimental infections caused the biggest negative impact on welfare. These animals are experimental and are being used to study the diseases aforementioned, as a result, clinical signs are the key feature of the studies they are involved in and this would have been taken into account in the harm: benefit assessment at the ethical review of the project license application. It is therefore expected that the clinical signs experienced by these animals will be thoroughly recorded, and they are likely to experience more severe clinical signs than most of their farm animal counterparts. Combined with the lack of available behavioural data, this may have skewed the importance of clinical assessment in these two species, which may be of reduced significance in non-experimental environments where animals are not being intentionally subjected to infectious diseases.

### 4.3. Housing Can Be Accurately Scored Using the AWAG and May Be Useful in Comparing the Quality of Different Housing Environments

Housing in both species remained constant throughout and could be scored accurately. Housing was scored from CCTV footage, along with information provided regarding housing dimensions and group sizes. This should also be relatively easy to score in non-experimental animals, where housing tends to be relatively constant. The AWAG could be a useful way of comparing the quality of different housing environments, as shown in previous studies [1].

### 4.4. Social Structure Was Difficult to Score Using the AWAG from the Data Available, and so Was Removed from the Results. It Could Be Argued Social Structure Is Not Essential for Welfare Assessment in Groups That Have Remained Stable for a Prolonged Period of Time

The social structure of these animals was difficult to score without constant video monitoring, which was not possible at this time. Dominant/submissive behaviour was not noticeable in any of the video footage clips seen but is likely to be subtle and require thorough long-term analysis. Once a hierarchy has established within both of these species overt aggression will reduce, making assessment more challenging once a group has settled [37,38]. It could be argued that scoring this factor when groups have remained stable for a long period of time is not necessarily key in assessing welfare, however, is likely to be useful and more practical to perform when groups are first mixed. As this could not be scored reliably in this paper, this factor was removed from the results, and as stated may not be essential to score unless groups are being newly introduced or mixed. 

### 4.5. Further Research Is Needed in the Potential of the AWAG for Aiding the Decision of When Euthanasia May Be Appropriate

The AWAG acts as a useful tool for monitoring how welfare might improve or deteriorate overtime, as well as a method of displaying this visually. As seen in the assessment timeline for porcine A (Figure 6), welfare score exponentially increased as the animal reached the humane end points set by the study. This shows strong potential in the AWAG being useful for helping to determine when euthanasia should be performed, especially in environments where end points are not specified. As an example, in a small animal with chronic progressive disease, regularly scoring these animals could help to demonstrate welfare deteriorating and identify when welfare has reached a point where euthanasia is the best option [39]. There is a need for further research in this area, as well as further testing of the application to other species.

### 4.6. The AWAG Was Successfully Adapted for Experimental Cattle and Pigs

The AWAG has already been successfully adapted and applied to a number of species, including zoo animals and primates [40]. This study demonstrated the potential use of the scoring system in farm animals, as it was easily adapted for experimental cattle and pigs. Further research would be useful in its applicability to farm animals in non-experimental conditions.

## 5. Conclusions

In summary, the AWAG can be successfully adapted and applied to cattle and pigs in a research environment, and further development of it is needed on to apply it to non-experimental farm animals. The AWAG would offer an opportunity to engage with farmers and the wider industry to motivate positive change. Animals with poor quality of life do not have normal physiology or behaviour and have been shown to produce poorer quality products and reduce economic benefit. Communicating welfare is challenging and societal concerns about livestock food animal production highlight the need for objective measurement of welfare in this sector. The AWAG would provide evidence of how improvements have been made and may be used to motivate those who produce, maintain or use animals to identify cases of sub-optimal quality of life, leading to better animal welfare and public perception and a good life, or a life worth living.

## Figures and Tables

**Table 1:** Table showing the full list of factors selected for both cattle and pigs, as well as the reasoning for selection.

Factor	Reasoning
Physical	
Body Condition score	Body condition scoring is a key measure of health and welfare state [19,20]. Weight loss is also a clinical sign of Foot and Mouth Disease (FMD) and African Swine Fever (ASF) [22,23].
Lameness score	Lameness in any animal is a sign that they are in pain, and a sign of ill-health and discomfort [19,20]. These are negative affective states and so need to be scored when assessing quality of life. Lameness is also a clinical sign of FMD and ASF [22,23]
Observable clinical signs (e.g., salivation, lethargy, pyrexia, diarrhoea, nasal discharge, etc.)	Maintenance of good health is the most basic requirement affecting welfare of cattle and pigs [19,20]. These animals are experimental and so are being subjected to infectious diseases. The physical effects of these must be assessed in order to determine the affect these diseases are having on physical health, and therefore welfare.
Food intake	
Presence of injury (not as a result of any procedure carried out by the study, e.g., kick injuries from other animals)	Contingent events (those that occur not as a direct result of the scientific study) must be factored into assessing welfare and quality of life [16]. Injuries causing pain and discomfort are classed as negative affective states and so will affect overall quality of life [24].
Procedural	
Impact of study procedures (including blood sampling, vaccine administration with sedation, rectal temperature and foot examination).	These animals are experimental and will be experiencing procedures carried out by the studies involved. Scientific procedures have the potential to cause pain, suffering, distress or harm and as a result the effect of these must be evaluated to assess quality of life [2].
Response to restraint for each of the above procedures.	In order for the above procedures to take place, some form of restraint is usually required. The response to this depends on how each particular animal perceives it, for example ‘one animal may be well habituated to restraint for examination and find restraint a positive experience with food treats, whilst another animal may be highly fearful and actively resist being restrained’. These animals will experience vastly different levels of fear-stress [25]. As a result, response to restraint will have a varying impact on each animal’s lifetime experience, and therefore needs to be assessed.
Psychological	
Display of abnormal/stereotypic behaviours	Abnormal behaviour is a ‘potential indicator of pain, suffering and injury’. Stereotypies, in particular, can be observed as a consequence of inadequate environmental conditions and impaired welfare [26].
Response to human activity (e.g., human presence during feeding/cleaning/routine daily inspection)	Human-animal interaction can have a profound impact on the welfare of farm animals. These interactions may be neutral, positive or negative in nature [27]. A good human-animal relationship is fundamental to farm animal welfare [28], and therefore needs to be included when assessing quality of life.
Use of enrichment provided	The benefits of providing enrichment will not be seen if the enrichment provided is not appropriate or is not being used. It is therefore important to assess use of enrichment as opposed to just the presence of enrichment.
Display of natural behaviours (species specific, e.g., various modes of locomotion, wallowing, ruminating, etc.)	Natural behaviours are behaviours that animals tend to exhibit under natural conditions, ‘because these behaviours are pleasurable and promote biological functioning’. Animals have a need to exercise certain natural behaviours, such as nest-building in pigs. All needs (not just physiological, such as the need for shelter, food and water) need to be taken into account in order to assess overall welfare [29].
Social structure (e.g., presence or absence of aggression/bullying/submissive behaviour)	Forming new groups can be stressful for farm animals. Regrouping destabilises the social dynamic which increases physical competition [30]. It is fair to assume bullying within the group will cause a negative emotional state in pigs and cattle. As a result, social structure and the relationships within a group should be assessed.
Environmental	
Housing (e.g., space provision, lighting, flooring, substrate, etc.)	The more limited the space that cattle and pigs have in a housing system, the less choice the animal has to avoid unfavourable conditions [19,20]. Indoor housing may compromise choice for the animal and restrict its freedom to express normal behaviour, e.g., zero outdoor grazing for cattle, and no access to wallows for swine [24]. This will have a negative impact on their welfare and so needs to be assessed.
Enclosure complexity (e.g., planting, water bodies, food, shelter, hiding places, ability to get away from other members of the group)	Accommodation should provide shelter and enough room to move around and interact with other individuals. This should include enough space for a subordinate animal to move away from a dominant one [19,20]. Enclosure complexity can contribute to negative and positive affective states, and so need to be factored into assessment.
Enrichment provision (species specific based on the DEFRA code of practice recommendations for each species)	Enrichment is a key aspect of animal welfare and reduces abnormal behaviours commonly seen in production animals [31]. The welfare of farmed pigs can be improved by modifying their environment with bedding, substrates or objects so they can perform more of their natural behaviours. Enrichment can also help manage undesirable and damaging behaviours such as tail biting, which if present will reduce quality of life [32]. Enrichment can also improve biological functioning, help animals cope with stressful surroundings, increase the fulfilment of behavioural needs and therefore promote more positive affective states [30]. Enrichment therefore has a significant impact on quality of life and provision needs to be assessed to ensure it is present and appropriate.
Group size	Group size in these herd animals affects social behaviour as well as stocking density and can therefore have a direct impact on welfare [24].

**Table 2:** Table showing all factors scored in cattle and pigs, separated into those that could be scored accurately using the available data, and those that could not be. The parameter each factor belongs to is shown in parenthesis.

Factors That Could Be Scored Accurately from the Available Retrospective Data.	Factors That Could Not Be Scored Accurately without Enhanced Data Collection during the Research Process.
Clinical signs (physical)	Natural behaviours (behavioural)
Food Intake (physical)	Response to catching event & human interaction (behavioural)
General Condition Score (physical)	Social structure (behavioural)
Lameness (physical)	Stereotypic behaviour (behavioural)
Presence of Injury (physical)	Interaction with enrichment (environmental)
Housing (environmental)	The effect of veterinary/husbandry procedures on welfare (procedural)
Group size (environmental)	The effect of restraint for regulated procedures on welfare (procedural)
Enclosure complexity (environmental)	
Study procedures (procedural)	

**Figure A1:**
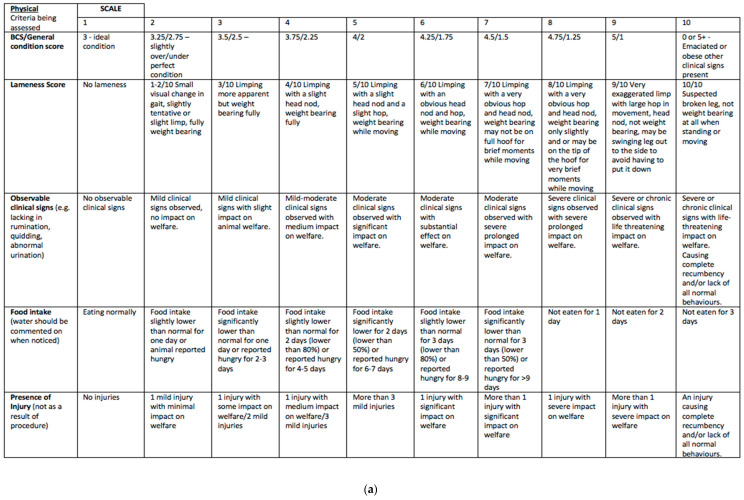

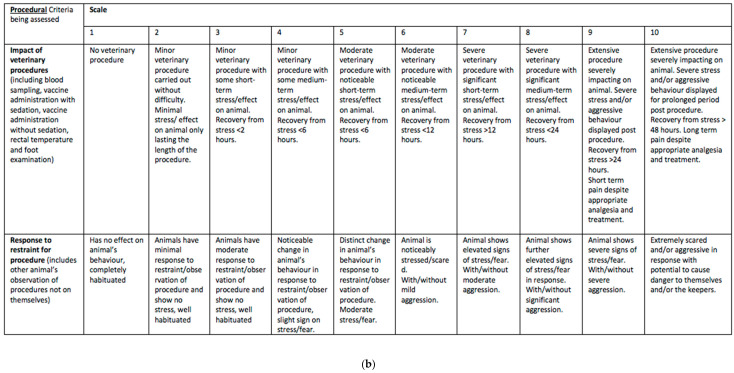

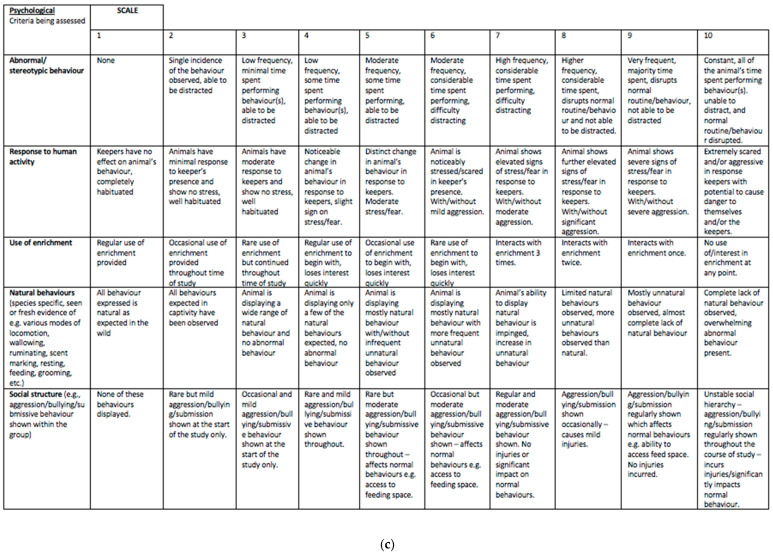

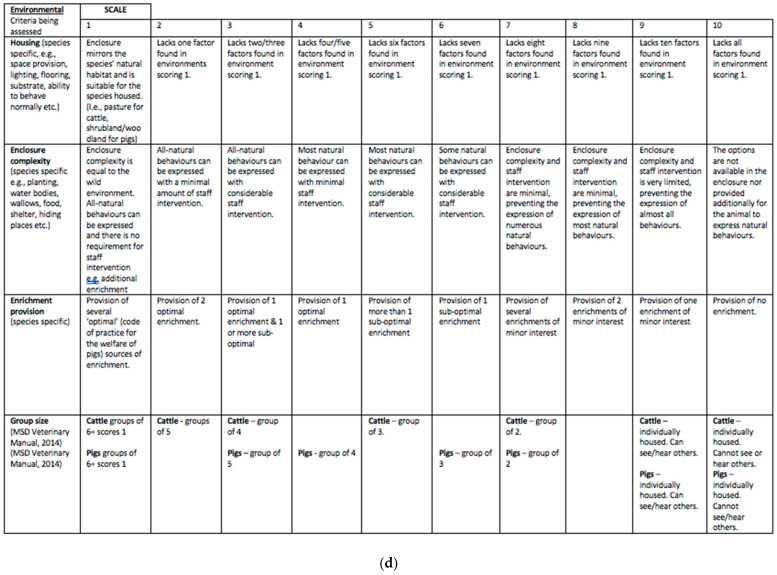
Criteria Tables for Each Factor. (**a**) Criteria table for scoring of physical factors. (**b**) Criteria table for scoring of procedural factors.; (**c**) Criteria table for scoring of psychological factors; (**d**) Criteria table for scoring of environmental factors.

## Data Availability

The anonymized raw data used in this study is available online at https://doi.org/10.6084/m9.figshare.13328807 (accessed on 4 March 21).

## Appendix A

**Table A1:** Criteria for Humane End Points (set by the respective FMD and ASF studies).

Humane End Points for Cattle Infected with Foot and Mouth Disease	Humane End Points for Pigs infected with African Swine Fever
Pyrexia—The animal will be euthanised if it has a fever above 40 °C at the beginning of the 4th consecutive day and has not responded to veterinary treatment. Lameness—The animal will be euthanised if not weight-bearing due to pain and inflammation from FMDV infection on one or more feet resulting in the affected animal showing reluctance to stand and/or if the lesions on the coronary band start to bleed at the beginning oft he 3rd consecutive day. Behaviour—The animal will be euthanised if it stays isolated, has a delayed response to stimuli, is slower in movement and lethargic, and/or presents an abnormal posture, e.g., back arched at the beginning of the 2nd consecutive day. Anorexia—The animal will be euthanised if it is not eating at the beginning of the 3rd consecutive day or sooner if the animal has any swelling of the tongue, face or oral lesions that prevent it from eating or drinking. The following signs are not typical of an uncomplicated case of FMD but could be seen with secondary infections or if aspiration pneumonia occurs due to the presence of oral lesions Respiratory System—The animal will be euthanised immediately if it shows respiratory distress or has an arched back with accompanying signs of fluid present on the lungs after chest auscultation. If the animal develops a persistent cough it will be euthanised at the beginning of the 3rd consecutive day. Digestive System—The animal will be euthanised if it has watery/haemorrhagic diarrhoea at the beginning of the 3rd consecutive day. Any animal showing 3 or more of the above clinical signs combined on a single day will be euthanised on the same day even if the duration of individual endpoints has not been reached.	Pyrexia—The animal will be euthanised if it has a fever above 40.5 °C and shows other clinical signs at the beginning of the third consecutive day. If the animal has no other clinical signs, apart from a raised temperature, it will be euthanised at the beginning of the fourth consecutive day of temperature. Behaviour—The animal will be euthanised if it has a tendency to stay isolated, shows a delayed response to stimuli (gets up slowly when touched), and/or presents an abnormal posture, e.g., head hung or back arched for two consecutive days. Anorexia—The animal will be euthanised if it is not eating on a second consecutive day. Digestive system—The animal will be euthanised if it has haemorrhagic diarrhoea. Respiratory system—Any pig showing an increase in breathing rate at the beginning of the second day without improvement will be euthanised at the beginning of the second day. Vomiting—The animal will be euthanised if vomiting is observed on the third day of pyrexia in association with one other of the above clinical signs. Lameness—Animals showing non-weight bearing lameness will be euthanised 48 h after treatment if it does not show improvement. Animals showing non-weight bearing lameness will be euthanised at the beginning of the 5^th^ consecutive day after treatment if lameness has not improved significantly. If there is a recurrence of the lameness, due to a regulated procedure, within 21 days from initial onset of lameness the animal will be euthanised on the same day. If the animal becomes lame due to a regulated procedure a third time, it will be euthanised on the same day. Any animal showing three or more of the above clinical signs combined on a single day will be euthanised on the same day even if the duration of individual endpoints has not been reached. If in any individual case it is considered necessary to maintain animals beyond these severity limits the Home Office Inspector will be consulted.
